# Fcγ receptor expression on monocytes and association with etanercept efficacy in rheumatoid arthritis: a prospective cohort study

**DOI:** 10.3389/fphar.2026.1807192

**Published:** 2026-04-24

**Authors:** Tongxin Wang, Hanchao Li, Li Zhu, Ying Pan, Jing Luo, Jing Zhang, Nan Hu, Lan He, Yining Sun

**Affiliations:** Department of Rheumatology and Immunology, The First Affiliated Hospital of Xi’an Jiaotong University, Xi’an, Shaanxi, China

**Keywords:** DAS28-CRP, DAS28-ESR, etanercept, Fcγ receptors, rheumatoid arthritis

## Abstract

**Background:**

To investigate whether Fcγ receptors (FcγRs) expression on peripheral blood monocytes predicts clinical response to etanercept in rheumatoid arthritis (RA).

**Methods:**

In this prospective cohort study, FcγRs expression on peripheral blood monocyte subsets was analyzed by flow cytometry. The primary outcome was the change in DAS28-CRP from baseline to week 12. The secondary outcome was the change in DAS28-ESR.

**Results:**

Following etanercept therapy, the proportion of FcγRI-positive monocytes and classical (CD14^++^CD16^−^) monocytes was reduced, while the proportion of non-classical (CD14^+^CD16^++^) monocytes was increased. Changes in intermediate monocytes (CD14^++^CD16^+^) were positively correlated with both DAS28-CRP change and DAS28-ESR change at week 24 after etanercept therapy. Patients with high intermediate (CD14^++^CD16^+^) monocytes showed significantly greater reductions in DAS28-CRP at week 12.

**Conclusion:**

Intermediate monocytes (CD14^++^CD16^+^) were correlated with improvements in DAS28-CRP achieved by week 12 following etanercept therapy. RA patients with low intermediate monocytes may derive less benefit from etanercept treatment.

## 1 Introduction

Rheumatoid arthritis (RA) is a chronic systemic autoimmune disorder characterized by persistent synovial inflammation, which triggers pain, swelling, and stiffness in the joint, and can progress to cause structural damage and deformity in both small and large joints (Gravallese and Firestein, 2023). Even with the advent of targeted biological disease-modifying anti-rheumatic drugs (DMARDs), which have markedly improved RA prognosis, approximately 40% of patients still respond poorly to treatment (Aletaha and Smolen, 2018; Konzett and Aletaha, 2024). Of note, the significant costs and potential adverse effects of DMARDs have emphasized the importance of identifying biomarkers capable of predicting treatment response, thereby enabling better patient stratification prior to therapy and improve treatment outcomes.

Tumor necrosis factor-α (TNF-α) inhibitors (TNFi) are the first-line biological DMARDs, achieving favorable therapeutic responses in 60%–70% of patients with RA (Ozen et al., 2021). TNFi function by inhibiting TNF-α, a pivotal pro-inflammatory cytokine that not only drives synovitis in active RA but also contributes to bone damage by synergistically promoting osteoclast differentiation and maturation. By effectively neutralizing TNF-α-mediated inflammation, TNFi treatment has been shown to significantly increase the lymphocyte-monocyte ratio and hemoglobin-red cell distribution width ratio, thereby ameliorating the chronic inflammation associated with RA, although these hematologic parameters do not predict treatment response (Yetişir et al., 2024). Etanercept, a representative TNFi, is a fusion protein composed of two recombinant human TNF receptor p75 monomeric chains linked to the Fc segment of human IgG1 (Feist et al., 2022). Etanercept exerts its therapeutic effects through high-affinity binding to soluble TNF-α (Kearsley-Fleet et al., 2018). However, evidence suggests that Fc gamma receptors (FcγRs) expressed on immune cells can specifically bind the Fc segment of etanercept, facilitating its cellular internalization and degradation, thereby influencing treatment efficacy (Hogarth, 2002; Mahmoud et al., 2023). We therefore hypothesize that variations in the binding affinity between FcγRs on immune cells and the Fc segment of etanercept could contribute to interindividual differences in responsiveness to etanercept.

FcγRs constitute a diverse class of glycoproteins expressed on the cell surface that serve as critical bridges between humoral and cellular immunity (Nimmerjahn and Ravetch, 2008). In humans, leukocytes express three classes of FcγRs: FcγRI, FcγRII (with isoforms IIa and IIb), and FcγRIII (including IIIa and IIIb) (Gillis et al., 2014). FcγRI, IIa and III trigger the activation of inflammatory cells via intracellular immunoreceptor tyrosine-based activation motif, whereas FcγRIIb crosslinking mediates suppression of these activating receptors through an intracellular immunoreceptor tyrosine-based inhibitory motif (Bruhns et al., 2009). Humans monocytes expressing FcγRIII are classified into three subsets according to their surface expression patterns of CD14 and CD16: classical (CD14^++^CD16^−^), intermediate (CD14^++^CD16^+^), and non-classical (CD14^+^CD16^++^) (Ziegler-Heitbrock et al., 2010). Notably, levels of FcγRI and FcγRII are upregulated in patients with active RA (Magnusson et al., 2014), and FcγRI-expressing monocytes were significantly reduced following etanercept treatment (Wijngaarden et al., 2008). Moreover, increased affinity for IgG1 has been shown to affect clinical and biological responses to rituximab, another type of biologic used in RA (Robinson et al., 2022). Nevertheless, the relationship between FcγR and response to etanercept in disease tissues remains unclear.

This prospective cohort study was designed to determine the correlation between changes in FcγR expression on peripheral blood mononuclear cells (PBMCs) and disease activity in patients with RA before and after etanercept treatment. Our findings revealed that the proportion of intermediate (CD14^++^CD16^+^) monocytes was significantly correlated with the change in Disease Activity Score for 28 joints based on the C-reactive protein level (DAS28-CRP) from baseline to week 12 following etanercept therapy. RA patients exhibiting a high proportion of intermediate (CD14^++^CD16^+^) monocytes are likely to experience greater therapeutic benefit from etanercept treatment.

## 2 Materials and methods

### 2.1 Study design and participants

This prospective cohort study was conducted in compliance with the Strengthening the Reporting of Observational Studies in Epidemiology (STROBE) reporting guidelines. The study enrolled patients from the Department of Rheumatology and Immunology at the First Affiliated Hospital of Xi’an Jiaotong University between July 2019 and June 2023.

Eligible patients received a subcutaneous dose of 50 mg etanercept once weekly, without any concomitant conventional synthetic disease-modifying antirheumatic drugs. The inclusion criteria were as follows: (1) age 18 years or over; (2) meeting the American College of Rheumatology/European League Against Rheumatism 2010 revised classification criteria for RA (Aletaha et al., 2010); (3) being in the active stage of the disease (DAS28-CRP score >=3.2); (4) willingness and ability to comply with scheduled visits, prescribed treatment plan, and laboratory assessments; and (5) capable of reading and understanding Chinese.

The exclusion criteria were as follows: (1) previous treatment with glucocorticoids and/or bisphosphonates for RA; (2) treatment with any biological agent (including TNFi, IL-6 receptor antagonist, or anti-CD20 monoclonal antibodies) within 6 months prior to enrollment; (3) comorbidity with other rheumatic autoimmune diseases (e.g., systemic lupus erythematosus or Sjögren’s syndrome); (4) concurrent infections (active tuberculosis, active hepatitis, or other active bacterial, viral, or fungal infections); and (5) history of malignancy and hematologic tumors.

Patients who failed to complete any items or altered their treatment plan during the follow-up period were excluded. This research was approved by the Ethics Committee of the First Affiliated Hospital of Xi’an Jiaotong University (Approval number: XJTU1AF2019LSL-019). All patients provided written informed consent.

### 2.2 Outcomes

The primary outcome was the change from baseline to week 12 in the DAS28-CRP score. The DAS28-CRP score ranges from 0 to 9.4, with higher scores reflecting greater disease activity. The DAS28-CRP score is calculated based on four variables: the tender and swollen joint counts (each out of 28 assessed joints); the patient’s global disease activity assessment (measured on a visual analogue scale ranging from 0 to 100 mm); and the serum high-sensitivity C-reactive protein level. The secondary outcome was the change in the Disease Activity Score for 28 joints based on the erythrocyte sedimentation rate (DAS28-ESR).

### 2.3 Flow cytometric analysis of FcγRs on PBMCs

PBMCs were isolated from heparinized blood via gradient centrifugation using Ficoll-Paque (GE Healthcare, Chicago, IL, USA) at 300 × g for 3 min. The isolated PBMCs were then washed twice with phosphate-buffered saline and resuspended in 800 μL of fluorescence-activated cell sorting buffer. Subsequently, PBMCs were stained with 10 μL of phycoerythrin (PE)-conjugated anti-CD14 antibodies (BD Pharmingen), in combination with one of the following fluorescein isothiocyanate (FITC)-labeled antibodies: 20 μL of anti-CD64 antibodies (BD Pharmingen), 10 μL of anti-CD32 antibodies (BD Pharmingen), or 10 μL of anti-CD16 antibodies (BD Pharmingen). FITC-conjugated anti-IgG1 kappa antibodies (Elabscience) were used as an isotype control. After washing with fluorescence-activated cell sorting washing buffer, PBMCs were resuspended and subjected to flow cytometry. FlowJo software was utilized for data analysis.

### 2.4 Sample size calculation

The sample size was calculated using G*Power (version 3.1) for a paired-samples *t*-test comparing DAS28-CRP scores between baseline and the 6-month follow-up. Based on an anticipated mean change of −2.7 units, a standard deviation of the change of 1.2, a two-sided alpha level of 0.01, and a desired power of 90%, a minimum of 7 participants was required. The target sample size was determined by factoring in a potential 20% attrition rate, and was accordingly set at 9 participants. Any cases with incomplete data were excluded from the final analysis.

### 2.5 Statistical analysis

Data collection was performed by experienced personnel to minimize measurement error. All data entries were cross-checked by two independent individuals to prevent data entry errors. Correlation between changes in FcγR expression on PBMCs and changes in DAS28-CRP or DAS28-ESR were analyzed using Pearson correlation analysis. Differences in baseline characteristics were analyzed using linear mixed-effects models for ANOVA (or Friedman test for non-normally distributed data). Differences in DAS28-CRP or DAS28-ESR changes after etanercept therapy between high and low FcγR expression groups were analyzed using either independent samples *t*-tests or the Mann-Whitney U test in cases of non-normal distribution. A two-sided alpha level of 0.05 was defined as the threshold for statistical significance. All analyses were performed using R (version 4.4.1) and SPSS (version 22.0).

## 3 Results

### 3.1 Patients and baseline characteristics

**FIGURE 1:**
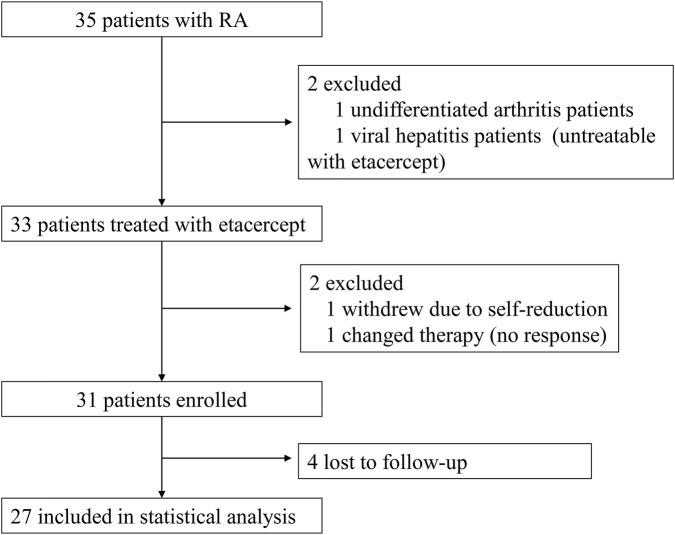
Study flow diagram.

Between July 2019 and June 2023, a total of 35 patients were screened for trial eligibility. One patient with undifferentiated arthritis and one with viral hepatitis were excluded prior to receiving etanercept. Among the remaining participants, one withdrew due to self-initiated dose reduction, one switched treatment due to lack of response, and four were lost to follow-up. Consequently, 27 patients were ultimately enrolled (Figure 1). The study population was predominantly female (82%), with a mean age of 44.85 ± 15.07 years and a median symptom duration of 3.00 (IQR: 2.00–6.00) years. At baseline, the mean DAS28-CRP score was 4.46 ± 0.93, indicating high disease activity. The median DAS28-ESR score was 4.44 (IQR: 3.95–5.30) (Table 1).

**TABLE 1:** Baseline and follow-up characteristics of patients.

Characteristics	RA patients (n = 27)			*F* value	*P* value
	Baseline	Follow-up (12 W)	Follow-up (24 W)		
Age (years)	44.85 ± 15.07			-	-
Females (%)	49 (82%)			-	-
Symptoms duration (years)	3.00 (2.00–6.00)			-	-
Rheumatoid factor (IU/mL)	99.95 (23.23–221.00)	73.90 (28.80–90.00)	67.40 (11.00–116.00)	9.333	0.009
ACPA (IU/mL)	338.10 (68.90–1,200.00)	421.20 (75.90–1,093.85)	182.00 (64.30–693.40)	4.791	0.091
ESR (mm/h)	30.00 (23.50–47.00)	16.00 (8.50–23.00)	15.94 ± 9.74	23.836	0.000
CRP (mg/L)	10.00 (5.82–14.60)	3.62 (0.58–10.00)	4.69 (0.76–10.00)	12.154	0.002
DAS28-ESR	4.44 (3.95–5.30)	2.75 ± 0.92	2.40 ± 0.95	46.110	0.000
DAS28-CRP	4.46 ± 0.93	2.92 ± 0.98	2.72 ± 0.91	30.710	0.000
FcγRI	64.71 ± 16.44	53.46 ± 15.03	52.82 ± 13.52	6.719	0.004
FcγRII	24.49 ± 13.83	20.59 ± 10.63	20.05 ± 13.65	3.982	0.031
Classical CD14^++^CD16^−^	69.44 ± 12.19	59.13 ± 14.78	55.09 ± 19.07	6.570	0.005
Intermediate CD14^++^CD16^+^	2.13 (1.43–3.47)	1.75 (1.10–2.91)	1.54 (1.20–3.47)	5.143	0.076
Non-classical CD14^+^CD16^++^	10.68 (6.59–12.66)	13.80 (9.05–19.88)	19.29 ± 8.30	6.182	0.006

ACPA, anti-citrullinated protein antibodies; CRP, C reactive protein; DAS28, 28-joint disease activity score; ESR, erythrocyte sedimentation rate; FcγRI, Fcγ receptors I; FcγRII, Fcγ receptors II; RA, rheumatoid arthritis. For normally distributed data, linear mixed-effects models for ANOVA, were used; For non-normally distributed data, Friedman test was used.

### 3.2 Expression of FcγRs on PBMCs after etanercept therapy

In patients with RA, the proportion of FcγRI-positive monocytes significantly decreased after 12 weeks (53.46% ± 15.03%) and 24 weeks of etanercept treatment (52.82% ± 13.52%) compared to baseline (64.71% ± 16.44%, *F* = 6.719, *P* = 0.004, Table 1). Although the proportion of FcγRII exhibited a downward trend at both 12 and 24 weeks compared to baseline, the difference was not statistically significant (*P* > 0.05). Additionally, etanercept treatment led to a significant reduction in the proportion of classical (CD14^++^CD16^−^) monocytes at 12 weeks (59.13% ± 14.78%) and 24 weeks (55.09% ± 19.07%) compared to the baseline levels (69.44% ± 12.19%, *F* = 6.570, *P* = 0.005, Table 1). In contrast, the proportion of non-classical (CD14^+^CD16^++^) monocytes exhibited a significant increase after both 12 and 24 weeks of etanercept treatment (Table 1). No statistically significant changes were observed in the proportion of intermediate (CD14^++^CD16^+^) monocytes at 12 or 24 weeks.

### 3.3 Correlation between changes in FcγRs expression and disease activity

**FIGURE 2:**
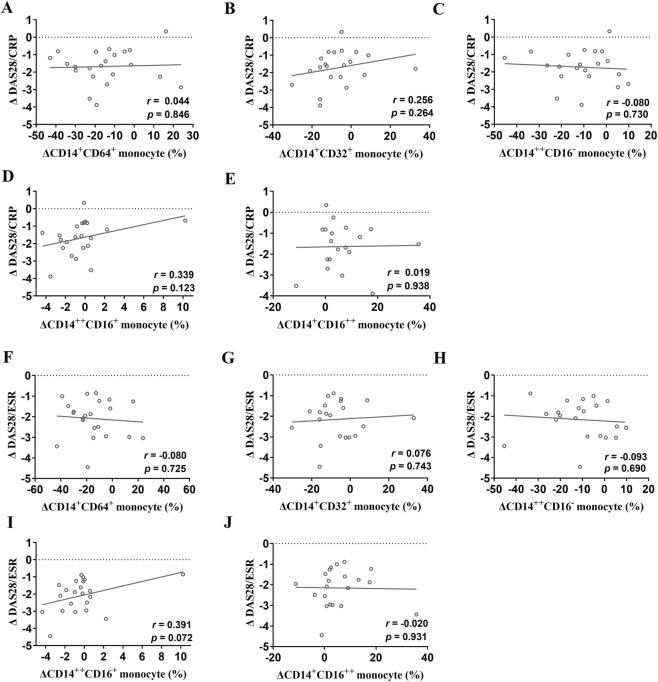
Correlations between changes in FcγR expression and changes in disease activity at week 12 after etanercept therapy. **(A–E)** Correlations between DAS28-CRP change and changes in FcγRI **(A)**, FcγRII **(B)**, classical (CD14^++^CD16^−^) monocytes **(C)**, intermediate (CD14^++^CD16^+^) monocytes **(D)**, or non-classical (CD14^+^CD16^++^) monocytes **(E)**; **(F–J)** Correlations between DAS28-ESR change and changes in FcγRI **(F)**, FcγRII **(G)**, classical (CD14^++^CD16^−^) monocytes **(H)**, intermediate (CD14^++^CD16^+^) monocytes **(I)**, or non-classical (CD14^+^CD16^++^) monocytes **(J)** at week 12 after etanercept therapy.

**FIGURE 3:**
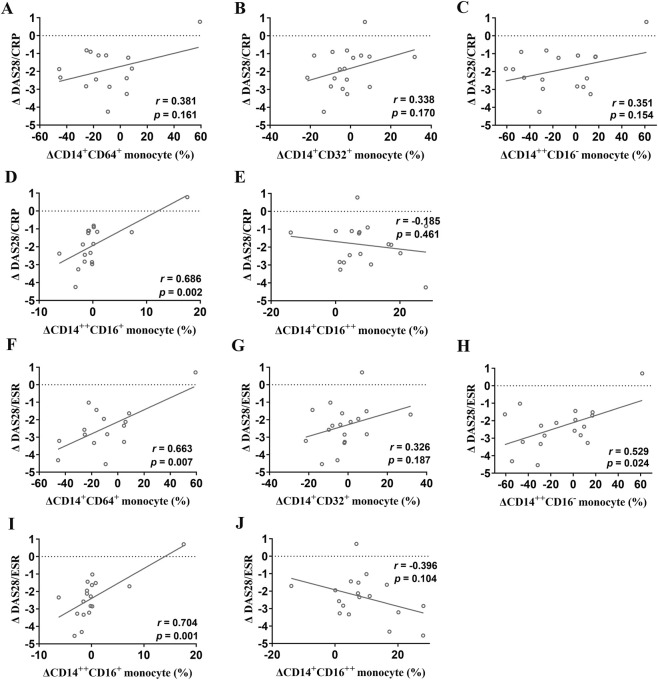
Correlations between changes in FcγRs expression and changes in disease activity at week 24 following etanercept therapy. **(A–E)** Correlations between DAS28-CRP change and changes in FcγRI **(A)**, FcγRII **(B)**, classical (CD14^++^CD16^−^) monocytes **(C)**, intermediate (CD14^++^CD16^+^) monocytes **(D)**, or non-classical (CD14^+^CD16^++^) monocytes **(E)** at week 24 following etanercept therapy; **(F–J)** Correlations between DAS28-ESR change and changes in FcγRI **(F)**, FcγRII **(G)**, classical (CD14^++^CD16^−^) monocytes **(H)**, intermediate (CD14^++^CD16^+^) monocytes **(I)**, or non-classical (CD14^+^CD16^++^) monocytes **(J)** at week 24 following etanercept therapy.

After 12 weeks of etanercept therapy, changes in FcγRI, FcγRII, and monocyte subsets (classical CD14^++^CD16^−^, intermediate CD14^++^CD16^+^, and non-classical CD14^+^CD16^++^) showed no significant correlation with the change in DAS28-CRP scores (Figures 2A–E). Similarly, no significant correlations were found between changes in DAS28-ESR and FcγR expression following 12 weeks of etanercept therapy (Figures 2F–J). In contrast, after 24 weeks of etanercept therapy, a significant positive correlation was identified between the change in intermediate (CD14^++^CD16^+^) monocytes and the change in DAS28-CRP (*r* = 0.686, *P* = 0.002; Figures 3A–E). The other two monocyte subsets, classical (CD14^+^CD16^−^) and non-classical (CD14^+^CD16^++^), showed no statistically significant relationship with the change in DAS28-CRP (Figures 3A–E). Additionally, changes in FcγRI expression were positively correlated with changes in DAS28-ESR (*r* = 0.663, *P* = 0.007; Figure 3F). Besides that, changes in classical (CD14^++^CD16^−^) monocytes (*r* = 0.529, *P* = 0.024) and intermediate (CD14^++^CD16^+^) monocytes (*r* = 0.704, *P* = 0.001) were positively correlated with changes in DAS28-ESR (Figures 3G–J).

### 3.4 Difference in DAS28-CRP and DAS28-ESR changes after etanercept therapy between high and low FcγR expression subgroups

RA Patients were assigned to either high or low FcγR expression subgroups based on median FcγR levels. Changes in DAS28-CRP and DAS28-ESR after etanercept therapy were compared between high and low FcγR subgroups. Reductions in DAS28-CRP at week 12 were significantly greater in the high intermediate (CD14^++^CD16^+^) subgroup compared with the low intermediate subgroup (*u* = 36.000, *P* = 0.035, Table 2). No significant differences in DAS28-CRP reduction were observed between high and low FcγR expression subgroups at 24 weeks after etanercept therapy (Table 2). Conversely, patients in the low classical (CD14^++^CD16^−^) monocytes subgroup achieved a significantly larger reduction in DAS28-ESR scores at week 12 compared to the high classical monocytes subgroup (*u* = 4.000, *P* = 0.014, Table 3). Similarly, a significant difference in DAS28-ESR reduction was observed at week 12 between the high and low intermediate (CD14^++^CD16^+^) monocytes subgroups (*t* = 2.915, *P* = 0.015). However, this difference was not maintained at week 24 (Table 3).

**TABLE 2:** The difference of DAS28-CRP change after etanercept therapy between high and low FcγR expression.

Group/statistic	ΔDAS28/CRP (12 w)	ΔDAS28/CRP (24 w)
FcγRI		
High expression	−2.28 ± 1.09	−2.28 ± 1.10
Low expression	−1.90 ± 1.05	−1.90 ± 1.05
*t (p*)	0.633 (0.540)	0.206 (0.841)
FcγRII		
High expression	−1.69 ± 1.19	−2.07 ± 1.19
Low expression	−1.84 ± 0.72	−2.15 ± 0.97
*t (p*)	0.286 (0.781)	0.118 (0.908)
Classical CD14^++^CD16^−^		
High expression	−0.83 (−1.44,-0.82)	−1.59 ± 0.78
Low expression	−2.48 (−2.83, −1.79)	−2.71 ± 1.04
*u*/*t (p*)	**4.000 (0.014)**	2.162 (0.058)
Intermediate CD14^++^CD16^+^		
High expression	−2.25 (−2.79,-1.51)	−2.59 ± 1.01
Low expression	−0.83 (−1.48, −0.81)	−1.54 ± 0.85
*u (p*)	**36.000 (0.035)**	2.042 (0.066)
Non-classical CD14^+^CD16^++^		
High expression	−1.69 ± 0.78	−2.01 ± 0.95
Low expression	−1.85 ± 1.22	−2.22 ± 1.23
*t (p*)	0.281 (0.785)	0.324 (0.753)

After removing one independent variable, changes in DAS28/CRP, were analyzed using independent samples t-tests (for normally distributed data) or Mann-Whitney U tests (for non-normally distributed data). Bold values indicate statistically significant differences (*p* < 0.05).

**TABLE 3:** The difference of DAS28-ESR change after etanercept therapy between high and low FcγR expression.

Group/statistic	ΔDAS28/ESR (12 w)	ΔDAS28/ESR (24 w)
FcγRI		
High expression	−2.49 (−3.24, −1.85)	−3.09 ± 1.14
Low expression	−2.75 (−2.97, −151)	−2.36 ± 0.77
*u*/*t (p*)	24.000 (0.731)	−1.364 (0.201)
FcγRII		
High expression	−1.81 (−2.99, −1.09)	−2.75 ± 1.36
Low expression	−2.97 (−3.02,-2.61)	−2.76 ± 0.53
*u*/*t (p*)	14.000 (0.367)	−0.029 (0.978)
Classical CD14^++^CD16^−^		
High expression	−1.96 ± 1.01	−2.54 ± 1.18
Low expression	−2.99 ± 0.84	−3.00 ± 0.92
*t (p*)	1.99 (0.072)	0.809 (0.435)
Intermediate CD14^++^CD16^+^		
High expression	−3.05 ± 0.79	−3.19 ± 0.98
Low expression	−1.72 ± 0.85	−2.25 ± 0.87
*t (p*)	**2.915 (0.015)**	−1.836 (0.094)
Non-classical CD14^+^CD16^++^		
High expression	−2.95 (−3.01,−2.19)	−2.67 ± 0.55
Low expression	−2.18 (−3.22,−1.21)	−2.85 ± 1.45
*u*/*t (p*)	24.000 (0.731)	−0.287 (0.784)

After removing one independent variable, changes in DAS28/ESR, were analyzed using independent samples *t*-tests (for normally distributed data) or Mann-Whitney *U* (for non-normally distributed data). Bold values indicate statistically significant differences (*p* < 0.05).

## 4 Discussion

In this prospective cohort study, etanercept treatment resulted in a reduction in FcγRI-positive monocytes and classical (CD14^++^CD16^−^) monocytes, as well as an increase in non-classical (CD14^+^CD16^++^) monocytes. Changes in intermediate (CD14^++^CD16^+^) monocytes were positively correlated with changes in both DAS28-CRP and DAS28-ESR at 24 weeks after etanercept therapy. Moreover, patients with higher baseline levels of intermediate monocytes exhibited significantly greater reductions in DAS28-CRP at week 12. Collectively, these findings provide evidence that RA patients with low levels of intermediate (CD14^++^CD16^+^) monocytes may benefit less from etanercept therapy.

FcγRs on immune cells can specifically bind to the Fc fragment of etanercept, promoting its cellular internalization and degradation, thereby influencing the drug’s efficacy (Hogarth, 2002; Mahmoud et al., 2023). Although previous studies have indicated that FcγR expression on monocytes may influence clinical and biological responses to TNFi in RA patients (Matt et al., 2015; Robinson et al., 2022), the relationship between peripheral blood monocyte FcγR expression and TNFi therapeutic efficacy remains poorly understood. Evidence suggests that nipocalimab, a neonatal Fc receptor-blocking monoclonal antibody, may offer therapeutic benefits, as observed in a population of anti-TNF inadequate responders with moderate to severe active seropositive RA (Taylor et al., 2024). Bazin et al. (2004) demonstrated that small tetramolecular immune complexes consisting of human Fc fragments and mouse anti-human IgG can impair the antibody-coated platelets clearance via interaction with low-affinity FcγRs. The present study found that etanercept treatment resulted in a decrease in FcγRI-positive monocytes and classical (CD14^++^CD16^−^) monocytes, and an increase in non-classical (CD14^+^CD16^++^) monocytes. In contrast, the proportion of FcγRII and intermediate (CD14^++^CD16^+^) monocytes was not significantly affected by etanercept therapy. These observations are consistent with prior reports indicating that the proportion of FcγRI-expressing monocytes decreases following etanercept treatment in RA patients, and that this reduction correlates with dampened inflammation (Wijngaarden et al., 2008). It has been reported that high expression of FcγRI is associated with disease activity (Luo et al., 2018). The equilibrium between activating and inhibitory FcγRs dictates the intensity of cellular immune responses, while dysregulation of this equilibrium contributes to the pathogenesis of autoimmune disorders, including RA and systemic lupus erythematosus (Wijngaarden et al., 2004; Carreño et al., 2009). Functional attributes of monocytes and macrophages, including phagocytic capacity and antigen-presenting function, are influenced by the equilibrium between activating and inhibitory Fcγ receptor signals (Clatworthy and Smith, 2004; Psaila and Bussel, 2008). Notably, therapeutic efficacy in RA often correlates with the restoration of FcγR balance (McGaha et al., 2005; Belostocki et al., 2008). The lack of change in FcγRII expression is likely due to opposing effects of TNF blockade (upregulating FcγRII) and disease improvement (downregulating FcγRII).

It has been reported that compared to healthy participants, patients with RA exhibited a reduced proportion of classical (CD14^++^CD16^−^) monocytes, while the proportions of both intermediate (CD14^++^CD16^+^) and non-classical (CD14^+^CD16^++^) monocytes were increased. The proportion of intermediate (CD14^++^CD16^+^) monocytes correlated positively with RA disease activity, whereas that of classical (CD14^++^CD16^−^) monocytes correlated negatively (Amoruso et al., 2016; Tsukamoto et al., 2017; Poole et al., 2024). Consistent with previous studies on TNFi (Batko et al., 2019), the present study found that etanercept treatment led to a decrease in classical (CD14^++^CD16^−^) monocytes and an increase non-classical (CD14^+^CD16^++^) monocytes. Notably, we observed that the change in intermediate (CD14^++^CD16^+^) monocytes was positively correlated with changes in both DAS28-CRP and DAS28-ESR at 24 weeks after etanercept therapy. Intermediate monocytes can differentiate into inflammatory macrophages, which promote inflammatory responses by secreting high levels of pro-inflammatory cytokines such as TNF-α, IL-6, and IL-8 (Rossol et al., 2012; Takai et al., 2015; Rana et al., 2018). According to Patel et al. (Patel et al., 2017), classical monocytes differentiate into intermediate monocytes, which in turn differentiate into non-classical monocytes, following a sequential ontogeny. This differentiation process enhances the cellular response to inflammatory signals. The decrease in classical monocytes, a non-significant reduction in intermediate monocytes, and a marked increase in non-classical monocytes indicate a shift toward a healthier phenotype, as confirmed in animal studies and RA patients, despite unchanged total monocyte counts (Shi and Pamer, 2011; Batko et al., 2019; Prajzlerová et al., 2021). Together with our findings, these results suggest that etanercept treatment may promote the conversion from classical and intermediate subsets to the non-classical subsets.

To ensure balanced patient allocation, trial participants were stratified according to baseline FcγR expression. At week 12, the subgroup with low classical (CD14^++^CD16^−^) monocytes achieved a significantly greater reduction in DAS28-CRP scores compared to the high classical monocytes subgroup. Similarly, a significantly greater reduction was observed in the high intermediate (CD14^++^CD16^+^) subgroup relative to the low intermediate subgroup. However, these differences were not sustained at week 24, as there were 23 sample sizes for DAS28-CRP assessment at week 12, and 18 at week 24, highlighting the need for larger studies to confirm this observation. These findings indicate that intermediate (CD14^++^CD16^+^) monocytes may serve as predictive indicators of a favorable response to etanercept in RA patients.

Some limitations exist within the design of this study. Although etanercept showed significantly greater efficacy at week 12, the short trial duration constrained the comprehensive assessment of outcomes and safety. The results at week 24 may have been influenced by the inclusion of patients with low disease activity, potentially introducing selection bias, and by the absence of a placebo control. The small sample size further limits the generalizability of our findings.

## 5 Conclusion

In this prospective cohort study of patients with RA receiving etanercept treatment, the change in intermediate (CD14^++^CD16^+^) monocytes was positively correlated with changes in both DAS28-CRP and DAS28-ESR at 24 weeks post-etanercept therapy. Reductions in DAS28-CRP at week 12 were significantly greater in high intermediate (CD14^++^CD16^+^) subgroup compared to the low intermediate subgroup, indicating that intermediate monocytes may serve as predictive indicators for a favorable response to etanercept in RA patients. Given the high cost of TNFi therapies, pre-treatment screening of RA patients by detecting FcγR expression levels on PBMCs could help identify those likely to respond well to therapy. Such an approach has the potential to alleviate the financial burden on patients, minimize unnecessary healthcare utilization, and offer substantial socioeconomic benefits. Furthermore, it provides a theoretical and experimental foundation for evaluating the efficacy of other TNFi in the treatment of RA.

## Data Availability

The original contributions presented in the study are included in the article, further inquiries can be directed to the corresponding author.
